# Lean thinking in health and nursing: an integrative literature review**^1^**

**DOI:** 10.1590/1518-8345.0979.2734

**Published:** 2016-08-08

**Authors:** Aline Lima Pestana Magalhães, Alacoque Lorenzini Erdmann, Elza Lima da Silva, José Luís Guedes dos Santos

**Affiliations:** 2 PhD, Adjunct Professor, Departamento de Enfermagem, Universidade do Estado de Santa Catarina, Chapecó, SC, Brazil.; 3 PhD, Full Professor, Departamento de Enfermagem, Universidade Federal de Santa Catarina, Florianópolis, SC, Brazil.; 4 PhD, Adjunct Professor, Departamento de Enfermagem, Universidade Federal do Maranhão, Florianópolis, SC, Brazil.; 5 PhD, Adjunct Professor, Departamento de Enfermagem, Universidade Federal de Santa Catarina, Florianópolis, SC, Brazil.

**Keywords:** Quality Management, Health Management, Efficiency, Organizational, Nursing, Health Systems

## Abstract

**Objectives:**

to demonstrate the scientific knowledge developed on lean thinking in health, highlighting the impact and contributions in health care and nursing.

**Method:**

an integrative literature review in the PubMed, CINAHL, Scopus, Web of Science, Emerald, LILACS and SciELO electronic library databases, from 2006 to 2014, with syntax keywords for each data base, in which 47 articles were selected for analysis.

**Results:**

the categories were developed from the quality triad proposed by Donabedian: structure, process and outcome. Lean thinking is on the rise in health surveys, particularly internationally, especially in the USA and UK, improving the structure, process and outcome of care and management actions. However, it is an emerging theme in nursing.

**Conclusion:**

this study showed that the use of lean thinking in the context of health has a transforming effect on care and organizational aspects, promoting advantages in terms of quality, safety and efficiency of health care and nursing focused on the patient.

## Introduction

The term, *lean thinking* (lean, lean thinking mentality, or lean thinking, in Portuguese) originated from the Toyota Production System. This term was first used by Krafcik and popularized by Womack and Jones, in 1992 with the publication of the book "the machine that changed the world"^1^ ^-^ ^2^. 

Despite the origin of lean thinking in the industrial context, its principles have been used in various scenarios, including health. This universal applicability of the lean concept is due to the similarity of the production processes of organizations, regardless of their specific nature, which try to plan and execute a series of actions in a certain sequence and time, to provide value for a customer ^3^ ^-^ ^4^. 

The introduction of lean thinking in health, or, lean healthcare, occurred in a structured and systematic way in 2006. That year, the Lean Enterprise Academy (LEA), a British non-profit, organization dedicated to the study and dissemination of lean thinking, organized the first congress on the implementation of lean principles in health services ^5^. 

Since then, health organizations have adopted lean thinking as a strategy to provide best care in several countries, including the United States-Thedacare (Wisconsin); Virginia Mason Medical Center (Washington), and Martin Health System (Florida); Sweden- Astrid Lindgren Children's Hospital; United Kingdom - the Bolton Hospitals; and Australia- the Flinders Medical Centre ^1^ ^,^ ^6^ ^-^ ^10^.

Lean within the health environment is still little explored in Brazil. During the search on Google Scholar, in 2014, five Brazilian studies were found: one article and four dissertations. The studies explore aspects of applicability and benefits of lean in a hospital laundry ^11^, the logistics activities of solid organ transplants ^12^, surgical center waste material ^13^, and the improvement of quality patient care and efficiency in health services ^5^. Only one publication reviewed identified areas, tools, methods and best practices in the implementation of lean concepts in hospital environments ^14^. Thus, lean health studies are incipient in Brazil, especially considering the magnitude of this issue in the international literature.

Lean thinking consists of a systematic approach that enables the identification and elimination of waste in production processes, focusing mainly on aggregate quality and delivering to the customer only what he considers to be of value ^15^. In other words, lean is the maximization of value for the client by means of an efficient process without waste. In health, this means providing services that respect and meet the needs and preferences of the patients ^10^. 

Another principle is the elimination of activities that do not generate value, along with any waste (long waits for care, duplicated actions, conflicting advice regarding treatment). Such waste does not allow that the process of care and treatment occur without interruption, detours, returns or delays. Thus, with the elimination of these issues, the efficiency of activities and quality of service simultaneously increase ^10^.

In the health service, the aspects that the patient values include a better, safer, faster, qualified and decisive care, according to his/her needs, aiming for the full recovery of his/her well-being ^16^ ^-^ ^17^. Health care improvement provided in all settings has occurred since the early days of hospital care, in order to improve the effectiveness of actions and provide quality support to the patients receiving these services ^18^. Lean thinking is a management model that has emerged as a reference for the scope of this quality care, combined with continuous improvement of the processes.

Several studies in the international literature about lean in health care aim to contribute to these scientific publications and innovations addressing this theme, therefore, the present study presents the contributions of this management model based on the triad of the quality evaluation model in health proposed by Donabedian ^(19)^. It is a widely diffused model within the quality health assessment area ^20^ and is directly connected to the search for continuous quality improvements, as well as lean thinking.

The three domains or conceptual assessment variables of this model are: structure, process and outcome. Structure is related to the physical and organizational settings, in which care occurs. Attributes included are: material resources (facilities, equipment and financial), human resources (quantity and qualifications of health professionals), and organizational structure (physical structure, organization of health care staff) ^19^ ^,^ ^21^ ^-^ ^23^. *Process* corresponds to the set of activities that occur between professionals and patients during care delivery and includes both the technical components of care (procedures, diagnosis and therapeutic interventions), as well as interpersonal relationships ^19^ ^,^ ^21^ ^-^ ^23^. *Outcome* refers to the effects of care on the patient's health status and also includes the customer and staff satisfaction with receiving and providing care, respectively ^19^ ^,^ ^21^ ^-^ ^23^. 

In light of the foregoing, the question is: what evidence is available in the scientific literature regarding the use of lean thinking in health care and nursing? How do lean practices impact the actions of health care in relation to the structure, process and outcome dimensions?

Thus, the objective was to demonstrate the scientific knowledge about lean thinking in the health area, emphasizing the impact and contributions to health care and nursing.

## Method

An integrative literature review was adopted to meet the objective of the study. This is a wider type of revision that includes both experimental and non-experimental research, allowing for the synthesis of multiple published studies and the development of a comprehensive explanation of a specific phenomenon. This method provides for identification of gaps in current knowledge that need to be understood with the development of new studies. The steps of this review were: statement of the research question; data collection; data evaluation; analysis and interpretation of data; presentation of results; and conclusions (24-25). 

**Figure 1:**
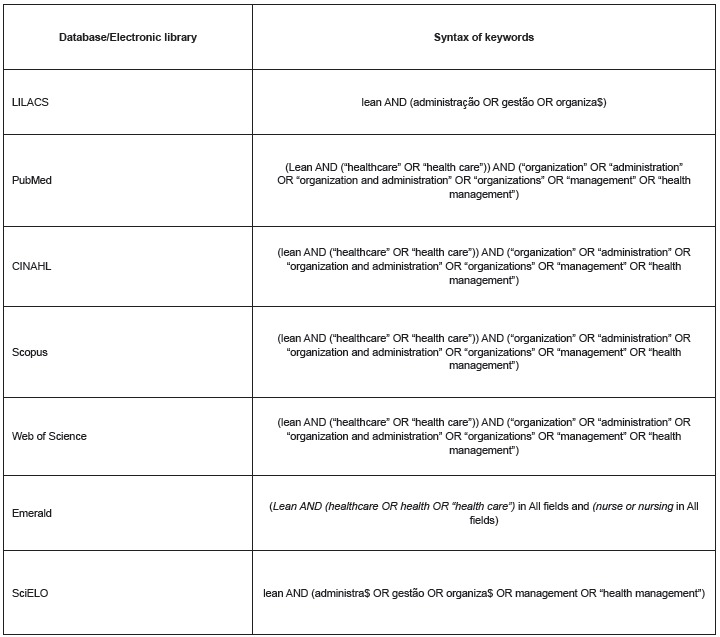
Syntax of keywords according to the databases. Florianópolis, SC, Brazil, in 2015.

Data collection was conducted in February and March of 2015, using: the Latin American and Caribbean Health Sciences (LILACS), Publisher Medline (PubMed), Cumulative Index to Nursing and Allied Health Literature (CINAHL), Scopus, Web of Science, Emerald and in the electronic library, Scientific Electronic Library Online (SciELO) databases. Figure 1 presents the syntax of the key words for searching the primary studies to ensure a comprehensive and reliable search. The keywords were used because standardized health descriptors for the studied subject do not exist yet.

Using these syntaxes, the following results were obtained: 26 articles in LILACS, 348 articles in PubMed, 181 articles in CINAHL, 567 in Scopus, 174 in Web of Science, 62 in Emerald, and 77 in SciELO, totaling 1,435 publications.

The inclusion criteria for articles were: original articles with abstracts available online, published from 2006 to 2014, in Portuguese, Spanish or English, that focus on lean thinking-related to health, as well as its contributions to the professional care actions of the area. This timeframe was used due to the fact that lean, in health, began to have greater visibility from the year 2006 (5). Articles duplicated in more than one database were deleted and only one was used. 

**Figure 2:**
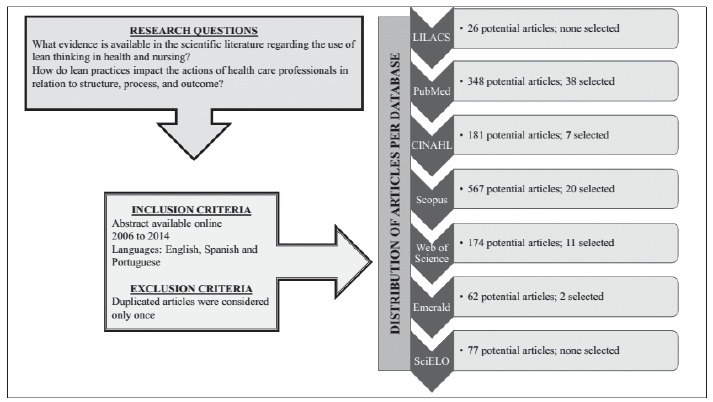
Flow diagram of research development. Florianópolis, SC, Brazil, 2015

The final sample consisted of 47 articles, PubMed (seven), CINAHL (seven), Scopus, (20), Web of Science (11), Emerald (2), as described in Figure 2. For the evaluation of studies, a data collection instrument was developed for information aiming to answer the question guiding the review. This instrument included the following items: identification of the study; goals, year and publication of journal; study design; and key findings and recommendations. The selected articles were analyzed and, to facilitate the organization of the data, we used the NVIVO(r) 10 software.

Data categorization was guided by the triad of a conceptual model for quality assessment in health, developed by Donabedian^19^. Aspects related to the physical structure of care environments, regulatory, technical and administrative, financial, training and availability of human resources and information system are considered as structural factors. In terms of process, the actions implemented effectively in the care environment, the changes implemented in care processes from the application of lean principles, and its tools used to improve the delivery of care were addressed. The outcomes indicated success, the benefits and the impact of lean practices in health, either for the patient or the health care provider, as well as the challenges to their implementation.

## Results

Based on the year of publication, an increase in new articles was observed, considering the 47 that were selected that were written over the years. The year of 2012 recorded the highest number of published articles, at 11 (23.4%).

Regarding publication language, all articles included were published in English, in 37 different journals, especially those related to management and quality in health care, such as the *Journal of Health Organization and Management* with four articles (8.5%); and two articles (5.9%) published in each of the following journals related to lean thinking in health: *BMC Health Services Research* with three articles (6.4%); *Quality & Safety in Health Care*; *Canadian Journal of Emergency Medical Care*; *International Journal of Health Care Quality Assurance*; *Leadership in Health Services*; *Quality & Safety in Health Care*; *Quality Management in Health Care*.

Regarding the country of origin of publication, 16 (34%) were submitted from the United States, seven (14.9%) from the United Kingdom, five (10.6%) from Holland, eight (17%) from Sweden, and two (4.3%) from Canada. Australia, Spain, Italy, Luxembourg, Malaysia, Norway, China accounted for another 14.9%, with one article for each country. Two studies (4.3%) were conducted in more than one country. No articles were identified from South America.

In relation to scenarios where the studies were conducted, the hospital environment was highlighted with 44 articles (93.6%). The most exploited sectors were: emergency rooms, operating rooms, and intensive care units. The other sites studied were primary care, with two articles (4.3%) and one article (2.1%) on both scenarios: hospitals and primary care.

Regarding the type of study, all articles included in the review were derived from original research; 28 (59.6%) used a quantitative approach, 12 (25.5%) a qualitative approach, and seven (14.9%) a mixed approach search. 

Another aspect analyzed was the profession of the authors. In this regard, of the 47 articles, 28 did not specify the professional background of the authors. Of the 19 who provided this information, it is emphasized that ten articles had nurse participation, four with the exclusive authorship coming from this profession.

**Figure 3:**
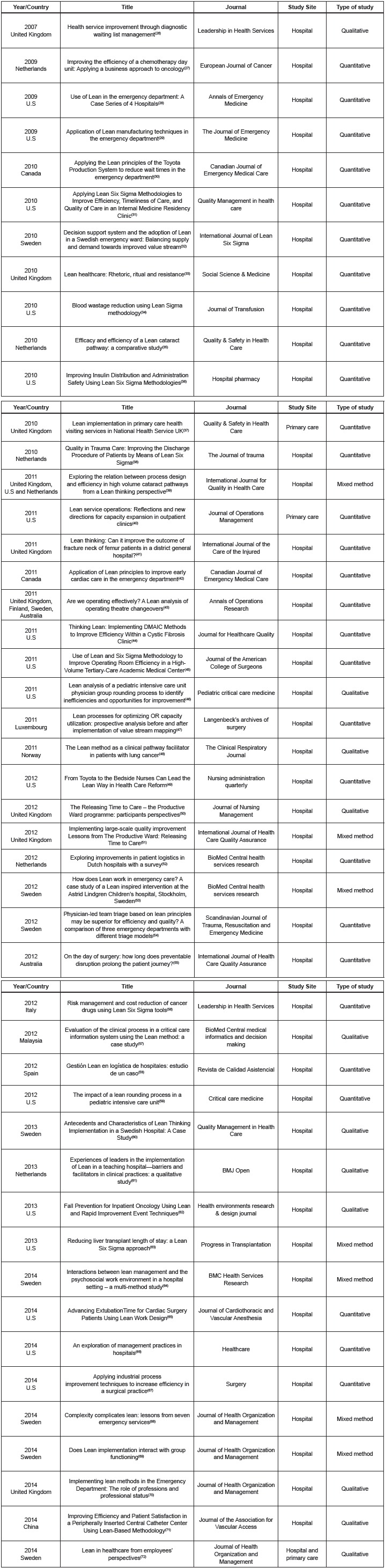
Characteristics of studies selected for the research. Florianópolis, SC, Brazil, 2015

Figure 3 shows the characteristics of the selected studies based on the year, country, title, publication journal, study setting, and type of study.

**Figure 4:**
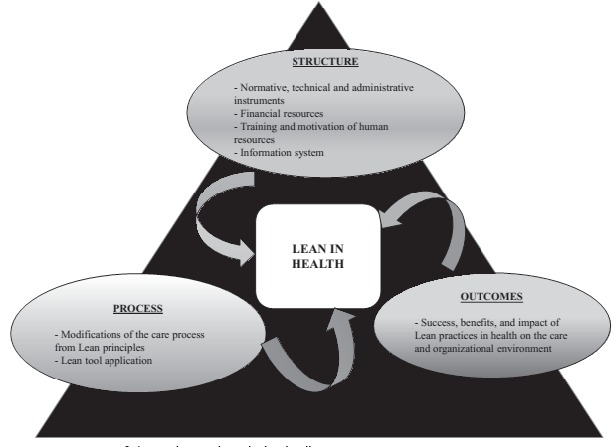
Summary of the main results. Florianópolis, 2015

Figure 4 summarizes the main results of the selected studies, based on Donabedian's quality triad.

## Discussion

The use of lean thinking is on the rise in health research in the global context and appears across multiple care settings. In this sense, it is observed that the articles were published in different journals which identify heterogeneity in the publication of the issue, but a homogeneous distribution of the number of articles published between them.

Most studies were implemented in the United States, which may be associated with the pioneering of this country in the implementation of lean concepts in health^(6.73)^.

With regard to the scenarios used for conducting these studies, the hospital environment predominated, across various units. This demonstrates the applicability of lean at a multiplicity of hospital care facilities. A similar result is described in a previous literature review on the implementation of lean thinking in healthcare^9^.

The incipient participation of nurses as authors on this subject was observed. This result may be associated with the origin of lean thinking in the area of administration and engineering^3^. 

Regarding the content of the articles, the results will be discussed according to the categories of quality assessment from the triad in health.

### Structure

Regarding the physical structure of the places where care is provided, the studies were implemented in various units, including: chemotherapy^27^, cardiology^60^, hospital pharmacy^36^, emergency^42^ ^,^ ^53^ ^,^ ^69^, intensive care^65^, and surgical centers^43^ ^,^ ^45^. It is noted that by modifying the layout of these environments using the lean approach, there was an ease of communication among the multidisciplinary team, resulting in increased efficiency and streamlining of patient care delivery.

Regarding the normative, technical and administrative instruments, lean thinking guides should be standardized and simplified care processes in order to reduce bureaucracy and facilitate health care. These changes inspired by lean reduce ambiguity at work, a more continuous flow of care, and allows team members to be more autonomous in the execution of activities and problem solving^36^ ^-^ ^37^ ^,^ ^53^ ^-^ ^56^ ^,^ ^66^ ^-^ ^67^. 

In a study performed in a surgical center^45^, a standardized description of the specific procedures of the surgeons allowed the health team to estimate the duration of each procedure. This enabled planning for the use of operating rooms in each specialty, helping to manage the need for opening and closing the rooms within each specialty. Another benefit of standardization of procedures was the ability to planning more precise surgical schedules, avoiding waste with incomplete or poorly distributed schedules.

In another study^43^ that examined elective orthopedic surgeries in five international hospitals, lack of standardization in processes was identified. The results showed that the standardization of processes in orthopedic surgery can improve productivity due to reduced variation in practice, affecting the efficiency of the surgical procedure time and patients waiting for this procedure, control of infection, and reduction in costs due to more efficient use of surgical rooms..

It is highlighted that the use of lean thinking provides economic improvements and positive impacts on the financial income of healthcare organizations, by increasing the capacity for patient care^27^ ^,^ ^44^ ^,^ ^52^ ^,^ ^67^ or allowing the reduction of financial costs due to the removal of fixed capital in the warehouses of health institutions^(36.56)^.

Another attribute of the structure component is human resources. By using lean to optimize the operating room capacity at a hospital in Luxembourg, it was possible to increase the number of annual surgeries without any increase in the quantity of personnel. However, this required training and motivation of the hospital staff, which had repercussions on eliminating unnecessary waiting periods and stress before surgery and increased the transfer rate of patients in the operating room to other units^47^.

The lean methodology is anchored in appreciation and respect for people, professional training and instructions on the workplace, enabling the improvement of professionals involved in the care process and optimizing the quality of care and patient safety^8^ ^-^ ^9^.

Research performed in the United States revealed that the ideal professional to conduct a lean transformation in a hospital is the nurse because he has experience leading multidisciplinary teams and is committed to patient care, and can view hospital systems from the patient's perspective^49^. However, the authors indicated that nursing education needs to be rethought and the curriculum should include concepts, tools, and skills necessary to adapt lean to the patient care environment. Organizational development, lean principles, quality improvement, inventory management, consulting process, value chain management, analysis queues, diffusion of innovation, complexity science and negotiation are some disciplines that must be aggregated into nursing curricula to prepare new nurses for the lean work in health institutions. Furthermore, in a study performed in Australia, it was identified that the principles of lean methodology have provided nurses greater satisfaction and control over their work^74^.

Information systems, also recognized as a component of structure, bring together a set of data, information, and knowledge to support the planning, development and decision-making processes of health professionals involved in patient care for the health system patients^75^.

A study performed in diagnostic support services in a clinical hospital in the United Kingdom (UK)^26^ used lean principles to manage the waiting list of patients awaiting diagnostic tests. The benefits identified by the study were the reduction in the waiting time for a diagnosis from 26 to 13 weeks, allowing for an early start of treatment. In addition, the study enabled increased control and better access to patient information, knowing who needed priority attention or who could remain on the waiting list for longer. Allied to this, managers were able to effectively manage the ability to meet demand because they came to understand the "profile" of waiting patients. Thus, there was an improvement in performance and quality of service offered to patients.

Another study performed in an emergency room in Sweden^32^ used a computer system for decision support, combined with lean as a tool to assist in making the best decisions. Through simulation, this program was considered and the potential effects of changes were evaluated by mimicking the behavior of a health system. The study showed that there was an imbalance in the number of clinicians and surgeons and the supply and demand of patients received in the emergency room, which caused excessive waiting time for the patients.

In addition to allowing the comparison between the current and future state in a particular care environment, the combination of simulation with lean thinking can make the flow of care more efficient, reducing the waiting list of patients and assisting in the selection of optimal resources, the process for service management in hospitals, eliminating or minimizing the use of a trial and error approach^76^.

### Process

For the care process to succeed there must be commitment, involvement and continued support of leaders for the "front line" professionals in the health institutions^26^ ^,^ ^28^ ^,^ ^60^ ^-^ ^61^ ^,^ ^64^ ^,^ ^68^ ^,^ ^70^ and a consideration of relevant ideas proposed by these workers^29^. In a study performed in the emergency unit, managers allowed the "front line" professionals to identify problems in patient flow in that sector and to reach their own solutions^29^. This positioning has improved the care provided to the patient and made sure the team was better able to create and introduce new ideas to solve the problems identified there, instead of having to implement guidelines that were imposed from the top down, by managers.

Lean thinking is the assumption that nurses and other professionals working on the "front line" of health services are better able to decide what patients need to have their needs met, considering good clinical practice^(8.74)^.

A study performed in a UK hospital specializing in cardiothoracic care highlighted the importance of managerial skills as essential strategies to facilitate the implementation of lean in health care, such as: open dialogue; ability to inspire people to approach old problems in new ways; maintaining the enthusiasm of workers; and the development of actions to facilitate and enhance communication among professionals ^50^.

The application of lean principles improves patient care. In a study performed in a hospital emergency room in Canada, for patients with acute coronary syndrome, it was possible to reduce the time of medical interpretation of the electrocardiogram (ECG), medical evaluation, and administration of acetylsalicylic acid through the use of lean principles^42^.

To achieve these goals some changes were necessary in the process of care for these patients, including: a specific ECG room next to the emergency triage was created; nurse triage is now the first contact for the patient in the unit, rather than the secretary; materials and equipment were relocated to reduce the movement of the nurse and the patient at the time of service; a procedure was established for immediate ECG interpretation by the doctor; readjustment of risk stratification tools for use by nurses; screening and clarification of the criteria for activation of the flowchart for care of patients with acute coronary syndrome. The application of lean principles significantly improved the performance of early diagnosis and therapeutic targets for emergency cardiac care in this service.

In addition to improving the care provided to patients, lean thinking is a method that seeks to understand the processes in order to identify and analyze problems and existing waste^34^ ^,^ ^46^ ^,^ ^53^ ^,^ ^57^ ^,^ ^59^ ^,^ ^65^; organizes the most effective and/or efficient processes ^46^ ^,^ ^48^ ^,^ ^59^; improves the detection of errors in these processes, providing information for their solution in order to avoid health damage^(36.55 to 56)^; and manages change and problem-solving using a scientific approach^46^ ^,^ ^53^ ^,^ ^58^.

Lean uses several tools to improve care processes, among which the following were highlighted in the articles: value stream mapping^26^ ^,^ ^28^ ^-^ ^30^ ^,^ ^32^ ^,^ ^37^ ^-^ ^38^ ^,^ ^41^ ^-^ ^42^ ^,^ ^44^ ^,^ ^46^ ^-^ ^47^ ^,^ ^49^ ^,^ ^53^ ^,^ ^55^ ^-^ ^57^ ^,^ ^59^ ^-^ ^60^ ^,^ ^64^ ^-^ ^65^ ^,^ ^71^; process mapping^(45.67)^; Kaizen^28^ ^-^ ^30^ ^,^ ^49^ ^,^ ^71^; standardization work^34^ ^,^ ^46^ ^-^ ^47^ ^,^ ^49^ ^,^ ^53^ ^,^ ^59^ ^,^ ^67^ ^-^ ^68^ ^,^ ^72^; a team approach to problem solving^28^; rapid improvement events^28^ ^,^ ^40^ ^,^ ^62^; pull system^41^ ^,^ ^47^; 5S^49^ ^,^ ^64^ ^,^ ^72^; A3^57^ and Kanban^58^. These tools enable the identification of waste and integrate stages of the most efficient and standardized processes.

## Outcomes 

All the articles analyzed reported successes and benefits for outcomes when using lean principles in health, both in health care and organizational aspects. The main effects found were: increased productivity and staff efficiency^27^ ^,^ ^32^ ^-^ ^33^ ^,^ ^36^ ^,^ ^40^ ^,^ ^43^ ^-^ ^45^ ^,^ ^47^ ^-^ ^48^ ^,^ ^50^ ^-^ ^52^ ^,^ ^55^ ^,^ ^58^ ^-^ ^59^; reduction in waiting time for patient care^26^ ^,^ ^30^ ^-^ ^31^ ^,^ ^33^ ^,^ ^39^ ^-^ ^40^ ^,^ ^42^ ^-^ ^43^ ^,^ ^45^ ^-^ ^50^ ^,^ ^55^ ^-^ ^56^ ^,^ ^63^ ^,^ ^65^ ^,^ ^67^; lower variability of care practices^33^ ^,^ ^36^ ^,^ ^39^ ^,^ ^43^ ^,^ ^46^ ^,^ ^53^ ^,^ ^57^; lower costs^29^ ^,^ ^34^ ^,^ ^36^ ^-^ ^37^ ^,^ ^39^ ^,^ ^56^; improved involvement and teamwork^43^ ^,^ ^45^ ^,^ ^47^ ^,^ ^51^ ^,^ ^53^ ^,^ ^57^ ^,^ ^60^ ^-^ ^61^ ^,^ ^64^ ^,^ ^70^; reduction in patient length of stay^28^ ^-^ ^30^ ^,^ ^38^ ^,^ ^54^ ^,^ ^63^, increased quality of service provided^(27 to 29.38)^, increased patient satisfaction^29^ ^-^ ^30^ ^,^ ^51^ ^,^ ^59^, increased use of hospital beds^(27 to 28.41)^; increased access to care^27^ ^,^ ^29^ ^,^ ^35^ ^,^ ^67^, increased patient safety and health professionals^36^ ^,^ ^56^ ^,^ ^62^, reduction in service errors ^55^ ^-^ ^56^, employee satisfaction^58^ ^-^ ^59^, reduction in mortality^(41.54)^, reduction in employee overtime ^(27.59)^; early hospital discharge^41^ ^,^ ^54^; and reduction in intubation time of patients^65^. In general, the application of lean in health benefits managers, health professionals, but especially patients. Similar data on the positive impact of this methodology have been found in other studies^4^ ^,^ ^73^.

However, in order for lean systems to improve metrics of care and patient satisfaction, health professionals, hospital managers, and the stakeholders involved will depend on the team's degree of commitment to lean principles and organizational culture^(28.61)^. It is essential that there is a continuous learning environment to facilitate the implementation of Lean principles. The challenges in implementing the Lean system are: to maintain the support of health professionals and leaders of hospitals, availability of time, resources and lack of training of leaders / managers with vision in Lean^30^ ^,^ ^33^ ^,^ ^35^ ^,^ ^45^ ^,^ ^49^. Similar barriers in the implementation of Lean were found in a study developed in Scotland^77^ and a narrative review^25^. To confront some of these challenges suggests showing the results of other places that have implemented Lean, especially those that directly affect employee satisfaction, as staff turnover and workload.

## Conclusion

This study enabled us to show that Lean thinking in health is a management model that improves the structure, process and outcome, from the care and management actions. The principles of Lean thinking is widespread in various contexts of health, such as emergency, oncology, pharmacy, intensive care unit, radiology, orthopedics, mental health clinics and cardiology services.

The main impacts from the application of this thinking in health are increasing productivity and team efficiency; reduction in waiting time for patient care; standardization of care process, reducing costs, improved teamwork, reduction in the patient's hospital length; increasing the quality of service provided; increased patient satisfaction; increasing patient safety and health professionals; and employee satisfaction.

It emphasizes the need for further studies on the applicability of Lean thinking in care environments in Brazil due to most publications focus on an international level, mainly in the United States and the United Kingdom. It is also important to highlight the importance of new nursing research that specifically objectifies the participation of the profession in this context. The participation of nurses in the scientific literature related to this issue is still incipient, even though he has been considered a professional able to lead a Lean transformation.
